# Supplementary material for: The Secretome of Irradiated Peripheral Mononuclear Cells Attenuates Hypertrophic Skin Scarring
Source: Pharmaceutics. 2023 Mar 25;15(4):1065. doi: 10.3390/pharmaceutics15041065 (PMC10143262; doi:10.3390/pharmaceutics15041065)
Supplement: Supplementary file 1 [file pharmaceutics-15-01065-s001.zip › pharmaceutics-2130546-supplementary.pdf]

Figure S1

(A)

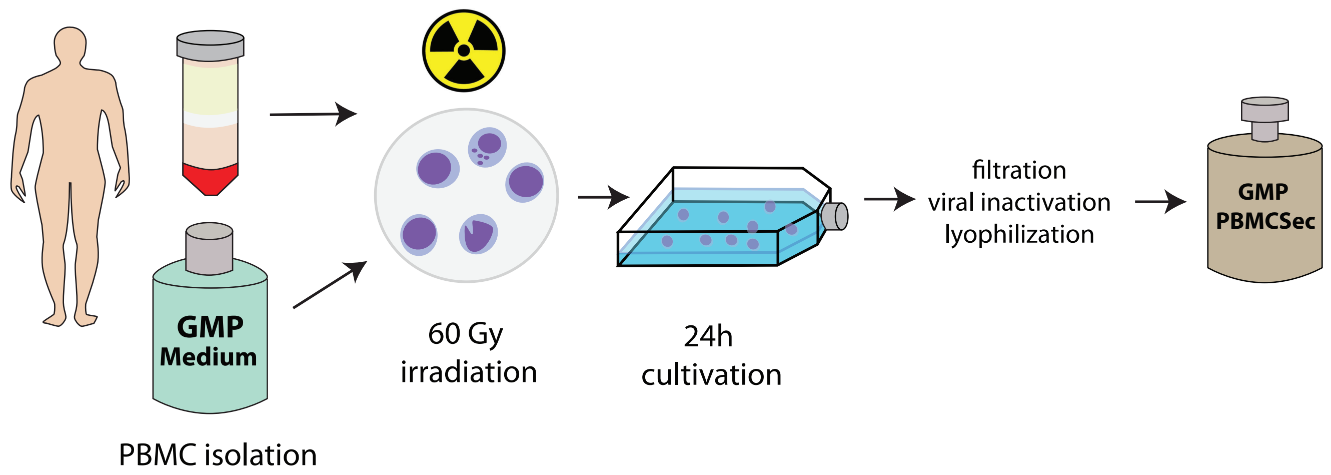

(B)

Target	Supplier	Product Nr.	Host species	Dilution	Application
GAPDH	abcam	ab8245	mouse monclonal	1:10000	WB
Collagen I	abcam	ab34710	rabbit polyclonal	1:200	IF
Collagen III	abcam	ab7778	rabbit polyclonal	1:200	IF
Elastin	Merck	Mab2503	mouse monclonal	1:100	IF, WB
Alexa fluor® 546 anti-mouse IgG (H + L)	Invitrogen	A-11030	goat polyclonal	1:500	IF, 2nd step
Alexa fluor® 546 anti-rabbit IgG (H + L)	Invitrogen	A-11035	goat polyclonal	1:500	IF, 2nd step
Anti-mouse, HRP-conjugated	GE Healthcar	GENX-A931	goat polyclonal	1:10 000	WB, 2nd step
Anti-rabbit, HRP-conjugated	Bio-Rad	#1706511	goat polyclonal	1:10 000	WB, 2nd step

Figure S1: Methods

A) Workflow of GMP-PBMCsec-production: leukocyte cones are obtained as blood-donation by-product, PBMCs are isolated by Ficoll-centrifugation, cells are irradiated with 60Gy and incubated for 24h. Superantatants are filtrated and lyophilized, and off-the-shelf PBMCsec is stored at -20° until use.
B) Table of antibodies used. IF = immunofluorescence, WB = western blot

Figure S2

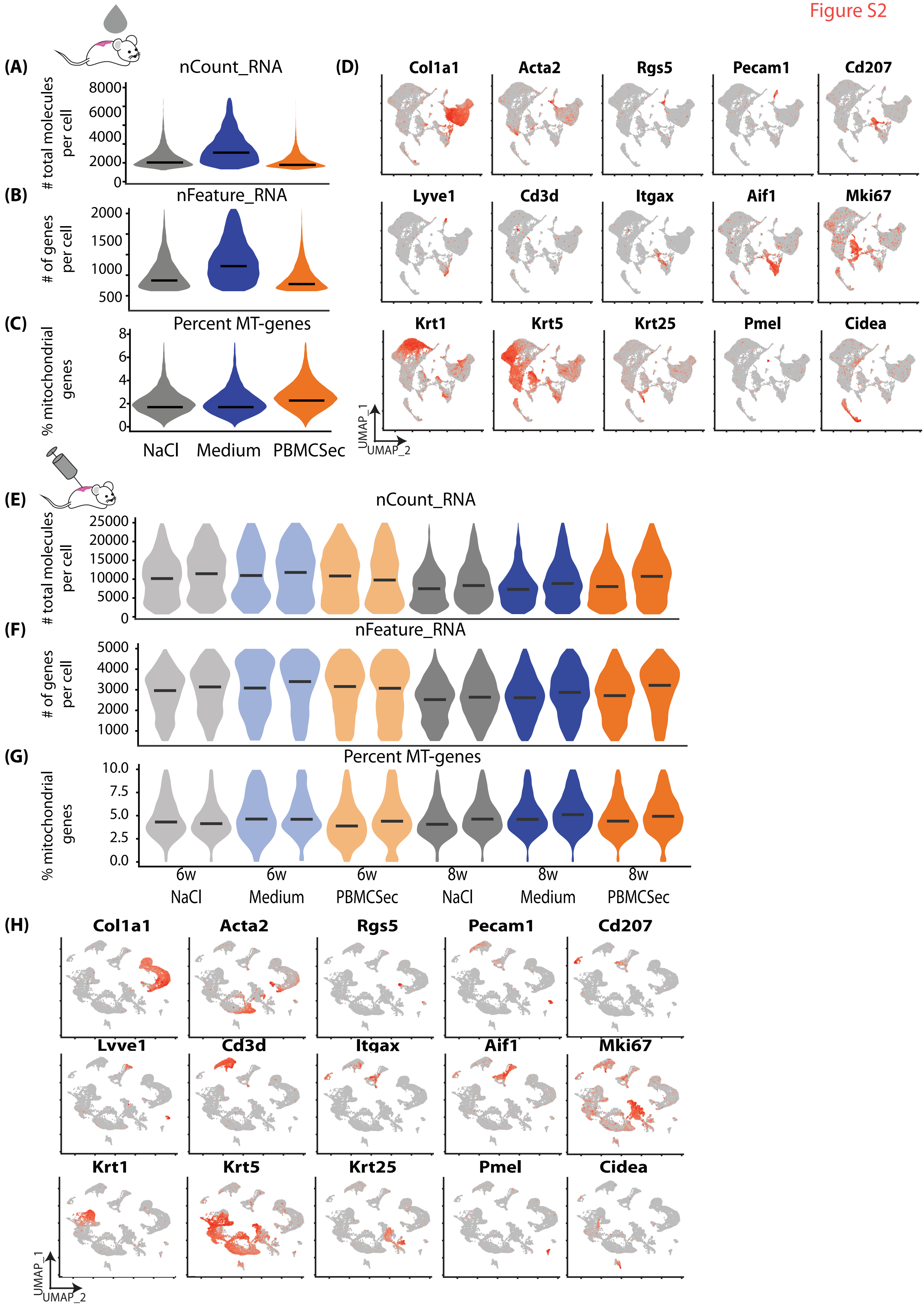
(A)
nCount_RNA
total molecules per cell
8000
6000
4000
2000
0
(B)
nFeature_RNA
# of genes per cell
2000
1500
1000
500
(C)
Percent MT-genes
% mitochondrial genes
8
6
4
2
0
NaCl
Medium
PBMCSec
(D)
Col1a1
Acta2
Rgs5
Pecam1
Cd207
Lyve1
Cd3d
Itgax
Aif1
Mki67
Krt1
Krt5
Krt25
Pmel
Cidea
UMAP_1
UMAP_2
(E)
nCount_RNA
total molecules per cell
25000
20000
15000
10000
5000
0
(F)
nFeature_RNA
# of genes per cell
5000
4000
3000
2000
1000
(G)
Percent MT-genes
% mitochondrial genes
10.0
7.5
5.0
2.5
0.0
6w NaCl
6w Medium
6w PBMCSec
8w NaCl
8w Medium
8w PBMCSec
(H)
Col1a1
Acta2
Rgs5
Pecam1
Cd207
Lyve1
Cd3d
Itgax
Aif1
Mki67
Krt1
Krt5
Krt25
Pmel
Cidea
UMAP_1
UMAP_2

Figure S2: Quality control of mouse scRNAseq

A-C, E-G) Violin plots of quality control parameters of the 'topical' and 'inject' dataset. A, E) total molecules per cell B, F) (gene count per cell and C, G) mitochondrial gene content. D, H) Feature Plots of cluster markers for cluster identification in the 'topical' and 'inject' dataset: *Col1a1* (collagen I alpha 1) for fibroblasts, *Acta2* (smooth muscle actin) for smooth muscle cells and myofibroblasts, *Rgs5* (Regulator Of G Protein Signaling 5) for pericytes, *Pecam* (Platelet And Endothelial Cell Adhesion Molecule 1) for endothelial cells, *Lyve1* (Lymphatic Vessel Endothelial Hyaluronan Receptor 1) for lymphatic endothelial cells, *Cd207* (Langerin) for Langerhans cells, *Cd3d* (cluster of differentiation 3D) for T-cells, *Itgax* for dendritic cells, *Aif1* (allograft inflammatory factor 1) for macrophages, *Mki67* (Marker Of Proliferation Ki-67) for proliferating cells, *Krt1* (Keratin1) for spinous and granular keratinocytes (KCs), *Krt5* (Keratin 5) for basal KCs, *Krt25* (Keratin 25) for hair follicles, *Pmel* (Premelanosome Protein) for melanocytes, *Cidea* (Cell Death Inducing DFFA Like Effector A) for adipocytes. Vertical lines in violin plots represent maximum expression, shape of each violin represents all results, and width of each violin represents frequency of cells at the respective expression level. In feature plots, normalized log expression of the respective gene is mapped onto the UMAP-plot. Color intensity indicates level of gene expressions. UMAP, uniform manifold approximation and projection.

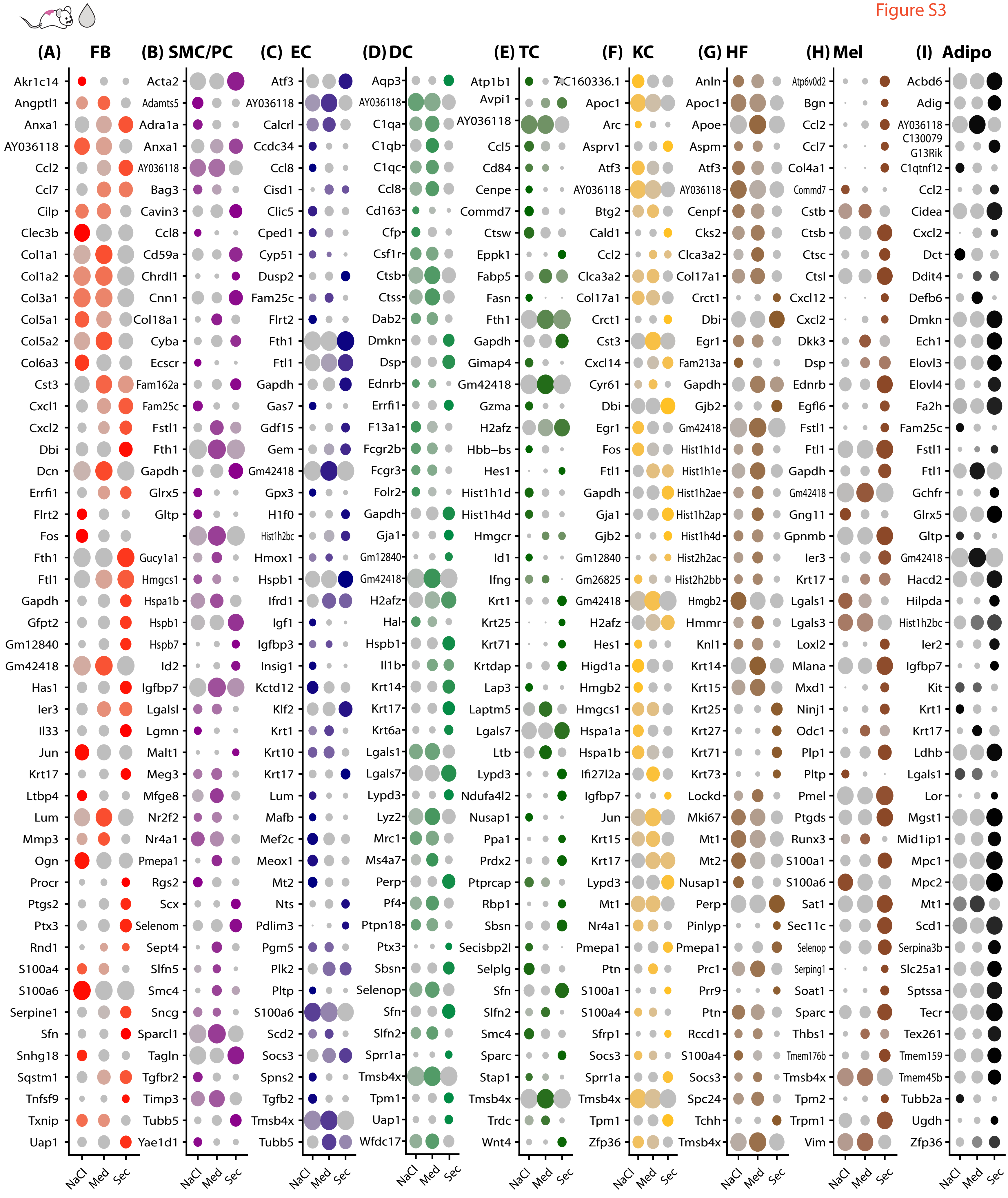
(A) FB
(B) SMC/PC
(C) EC
(D) DC
(E) TC
(F) KC
(G) HF
(H) Mel
(I) Adipo
NaCl
Med
Sec

Figure S3: **Top 50 regulated genes per cell group in 'Topical' mouse scars**

In A) fibroblasts (FBs, red circles), B) smooth muscle cells and pericytes (SMC/PCs, purple), C) endothelial cells (ECs, blue), D) T-cells (dark green), E) dendritic cells and macrophages (DCs, light green), and keratinocytes (KCs, yellow), hair follicles (HF, beige), melanocytes (Mel, brown), adipocytes (Adipo, grey); differentially expressed genes (DEGs) were calculated comparing 'PBMCsec' mouse scars to 'NaCl'-mouse scars, using Wilcoxon rank sum test, including genes with average logarithmic fold change (avglogFC) of > 0.1 or < -0.1 and Bonferroni-adjusted p-value <0.05. For each cellgroup, top 50 DEGs according to lowest adjusted p-value are displayed, split by treatment. Dot size represents percent of cells expressing the respective gene, color correlates with average expression.

Figure S4

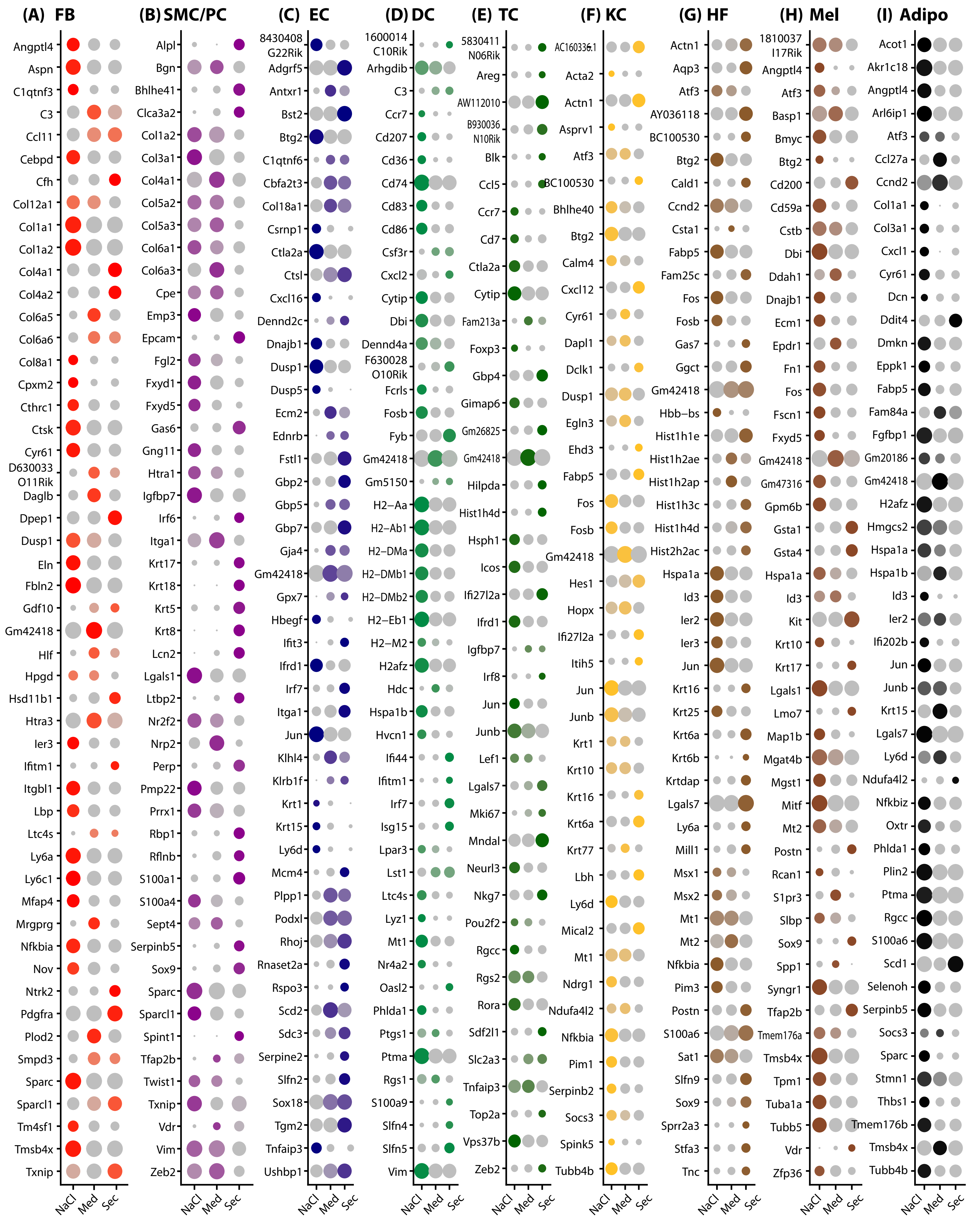

(A) FB
Angptl4
Aspn
C1qtnf3
C3
Ccl11
Cebpd
Cfh
Col12a1
Col1a1
Col1a2
Col4a1
Col4a2
Col6a5
Col6a6
Col8a1
Cpxm2
Cthrc1
Ctsk
Cyr61
D630033 O11Rik
Daglb
Dpep1
Dusp1
Eln
Fbln2
Gdf10
Gm42418
Hlf
Hpgd
Hsd11b1
Htra3
Ier3
Ifitm1
Itgbl1
Lbp
Ltc4s
Ly6a
Ly6c1
Mfap4
Mrgprg
Nfkbia
Nov
Ntrk2
Pdgfra
Plod2
Smpd3
Sparc
Sparcl1
Tm4sf1
Tmsb4x
Txnip
(B) SMC/PC
Alpl
Bgn
Bhlhe41
Clca3a2
Col1a2
Col3a1
Col4a1
Col5a2
Col5a3
Col6a1
Col6a3
Cpe
Emp3
Epcam
Fgl2
Fxyd1
Fxyd5
Gas6
Gng11
Htra1
Igfbp7
Irf6
Itga1
Krt17
Krt18
Krt5
Krt8
Lcn2
Lgals1
Ltbp2
Nr2f2
Nrp2
Perp
Pmp22
Prrx1
Rbp1
Rflnb
S100a1
S100a4
Sept4
Serpinb5
Sox9
Sparc
Sparcl1
Spint1
Tfap2b
Twist1
Txnip
Vdr
Vim
Zeb2
(C) EC
8430408 G22Rik
Adgrf5
Antxr1
Bst2
Btg2
C1qtnf6
Cbfa2t3
Col18a1
Csrnp1
Ctla2a
Ctsl
Cxcl16
Dennd2c
Dnajb1
Dusp1
Dusp5
Ecm2
Ednrb
Fstl1
Gbp2
Gbp5
Gbp7
Gja4
Gm42418
Gpx7
Hbegf
Ifit3
Ifrd1
Irf7
Itga1
Jun
Klhl4
Klrb1f
Krt1
Krt15
Ly6d
Mcm4
Plpp1
Podxl
Rhoj
Rnaset2a
Rspo3
Scd2
Sdc3
Serpine2
Slfn2
Sox18
Tgm2
Tnfaip3
Ushbp1
(D) DC
1600014 C10Rik
Arhgdib
C3
Ccr7
Cd207
Cd36
Cd74
Cd83
Cd86
Csf3r
Cxcl2
Cytip
Dbi
Dennd4a
F630028 O10Rik
Fcrls
Fosb
Fyb
Gm42418
Gm5150
H2−Aa
H2−Ab1
H2−DMa
H2−DMb1
H2−DMb2
H2−Eb1
H2−M2
H2afz
Hdc
Hspa1b
Hvcn1
Ifi44
Ifitm1
Irf7
Isg15
Lpar3
Lst1
Ltc4s
Lyz1
Mt1
Nr4a2
Oasl2
Phlda1
Ptgs1
Ptma
Rgs1
S100a9
Slfn4
Slfn5
Vim
(E) TC
5830411 N06Rik
Areg
AW112010
B930036 N10Rik
Blk
Ccl5
Ccr7
Cd7
Ctla2a
Cytip
Fam213a
Foxp3
Gbp4
Gimap6
Gm26825
Gm42418
Hilpda
Hist1h4d
Hsph1
Icos
Ifi27l2a
Ifrd1
Igfbp7
Irf8
Jun
Junb
Lef1
Lgals7
Mki67
Mndal
Neurl3
Nkg7
Pou2f2
Rgcc
Rgs2
Rora
Sdf2l1
Slc2a3
Tnfaip3
Top2a
Vps37b
Zeb2
(F) KC
AC160336.1
Acta2
Actn1
Asprv1
Atf3
BC100530
Bhlhe40
Btg2
Calm4
Cxcl12
Cyr61
Dapl1
Dclk1
Dusp1
Egln3
Ehd3
Fabp5
Fos
Fosb
Gm42418
Hes1
Hopx
Ifi27l2a
Itih5
Jun
Junb
Krt1
Krt10
Krt16
Krt6a
Krt77
Lbh
Ly6d
Mical2
Mt1
Ndrg1
Ndufa4l2
Nfkbia
Pim1
Serpinb2
Socs3
Spink5
Tubb4b
(G) HF
Actn1
Aqp3
Atf3
AY036118
BC100530
Btg2
Cald1
Ccnd2
Csta1
Fabp5
Fam25c
Fos
Fosb
Gas7
Ggct
Gm42418
Hbb−bs
Hist1h1e
Hist1h2ae
Hist1h2ap
Hist1h3c
Hist1h4d
Hist2h2ac
Hspa1a
Id3
Ier2
Ier3
Jun
Krt16
Krt25
Krt6a
Krt6b
Krtdap
Lgals7
Ly6a
Mill1
Msx1
Msx2
Mt1
Mt2
Nfkbia
Pim3
Postn
S100a6
Sat1
Slfn9
Sox9
Sprr2a3
Stfa3
Tnc
(H) Mel
1810037 I17Rik
Angptl4
Atf3
Basp1
Bmyc
Btg2
Cd200
Cd59a
Cstb
Dbi
Ddah1
Dnajb1
Ecm1
Epdr1
Fn1
Fos
Fscn1
Fxyd5
Gm42418
Gm47316
Gpm6b
Gsta1
Gsta4
Hspa1a
Id3
Kit
Krt10
Krt17
Lgals1
Lmo7
Map1b
Mgat4b
Mgst1
Mitf
Mt2
Postn
Rcan1
S1pr3
Slbp
Sox9
Spp1
Syngr1
Tfap2b
Tmem176a
Tmsb4x
Tpm1
Tuba1a
Tubb5
Vdr
Zfp36
(I) Adipo
Acot1
Akr1c18
Angptl4
Arl6ip1
Atf3
Ccl27a
Ccnd2
Col1a1
Col3a1
Cxcl1
Cyr61
Dcn
Ddit4
Dmkn
Eppk1
Fabp5
Fam84a
Fgfbp1
Gm20186
Gm42418
H2afz
Hmgcs2
Hspa1a
Hspa1b
Id3
Ier2
Ifi202b
Jun
Junb
Krt15
Lgals7
Ly6d
Ndufa4l2
Nfkbiz
Oxtr
Phlda1
Plin2
Ptma
Rgcc
S100a6
Scd1
Selenoh
Serpinb5
Socs3
Sparc
Stmn1
Thbs1
Tmem176b
Tmsb4x
Tubb4b
NaCl
Med
Sec

Figure S4: Top 50 regulated genes per cell group in 6 weeks 'Inject' mouse scars

In A) fibroblasts (FBs, red circles), B) smooth muscle cells and pericytes (SMC/PCs, purple), C) endothelial cells (ECs, blue), D) T-cells (dark green), E) dendritic cells and macrophages (DCs, light green), and keratinocytes (KCs, yellow), hair follicles (HF, beige), melanocytes (Mel, brown), adipocytes (Adipo, grey); differentially expressed genes (DEGs) were calculated comparing 'PBMCsec' mouse scars to 'NaCl'-mouse scars, using Wilcoxon rank sum test, including genes with average logarithmic fold change (avglogFC) of > 0.1 or < -0.1 and Bonferroni-adjusted p-value <0.05. For each cellgroup, top 50 DEGs according to lowest adjusted p-value are displayed, split by treatment and timepoint. Dot size represents percent of cells expressing the respective gene, color correlates with average expression.

Figure S5

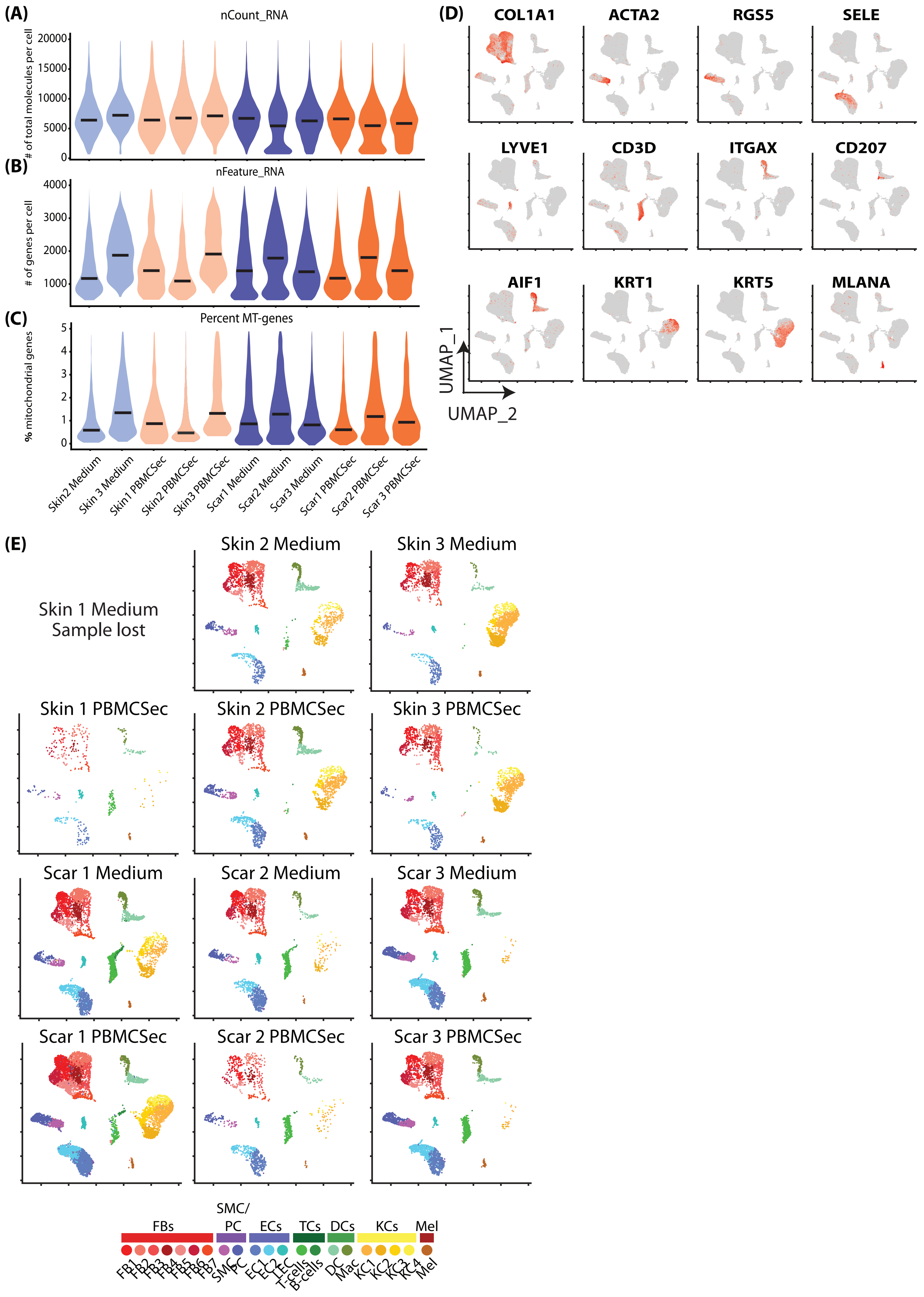
(A)
nCount_RNA
# of total molecules per cell
20000
15000
10000
5000
0
(B)
nFeature_RNA
# of genes per cell
4000
3000
2000
1000
(C)
Percent MT-genes
% mitochondrial genes
5
4
3
2
1
0
Skin2 Medium
Skin 3 Medium
Skin1 PBMCSec
Skin2 PBMCSec
Skin3 PBMCSec
Scar1 Medium
Scar2 Medium
Scar3 Medium
Scar1 PBMCSec
Scar2 PBMCSec
Scar 3 PBMCSec
(D)
COL1A1
ACTA2
RGS5
SELE
LYVE1
CD3D
ITGAX
CD207
AIF1
KRT1
KRT5
MLANA
UMAP_1
UMAP_2
(E)
Skin 1 Medium
Sample lost
Skin 2 Medium
Skin 3 Medium
Skin 1 PBMCSec
Skin 2 PBMCSec
Skin 3 PBMCSec
Scar 1 Medium
Scar 2 Medium
Scar 3 Medium
Scar 1 PBMCSec
Scar 2 PBMCSec
Scar 3 PBMCSec
FBs
SMC/ PC
ECs
TCs
DCs
KCs
Mel
FB1 FB2 FB3 FB4 FB5 FB6 FB7
SMC PC
EC1 EC2 LEC
T-cells B-cells
DC Mac
KC1 KC2 KC3 KC4
Mel

Figure S5: Quality control of human scRNAseq

A-C) Violin plots of quality control parameters of the 'human' dataset. A) total molecules per cell B) (gene count per cell and C) mitochondrial gene content. D) Feature Plots of cluster markers for cluster identification in the 'topical' and 'inject' dataset: *COL1A1* (collagen I alpha 1) for fibroblasts, *ACTA2* (smooth muscle actin) for smooth muscle cells and myofibroblasts, *RGS5* (Regulator Of G Protein Signaling 5) for pericytes, *SELE* (Selectin E) for endothelial cells, *LYVE1* (Lymphatic Vessel Endothelial Hyaluronan Receptor 1) for lymphatic endothelial cells, *Cd3d* (cluster of differentiation 3D) for T-cells, *ITGAX* for dendritic cells, *CD207* (Langerin) for Langerhans cells, *AIF1* (allograft inflammatory factor 1) for macrophages, *KRT1* (Keratin1) for spinous and granular keratinocytes (KCs), *KRT5* (Keratin 5) for basal KCs, *MLANA* (Melan-A) for melanocytes. E) UMAP-clustering in the 'human' dataset, split by samples. Sample "Skin 1 medium" was lost due to technical difficulties during preparation. Vertical lines in violin plots represent maximum expression, shape of each violin represents all results, and width of each violin represents frequency of cells at the respective expression level. In feature plots, normalized log expression of the respective gene is mapped onto the UMAP-plot. Color intensity indicates level of gene expressions. UMAP, uniform manifold approximation and projection

Figure S6 skin

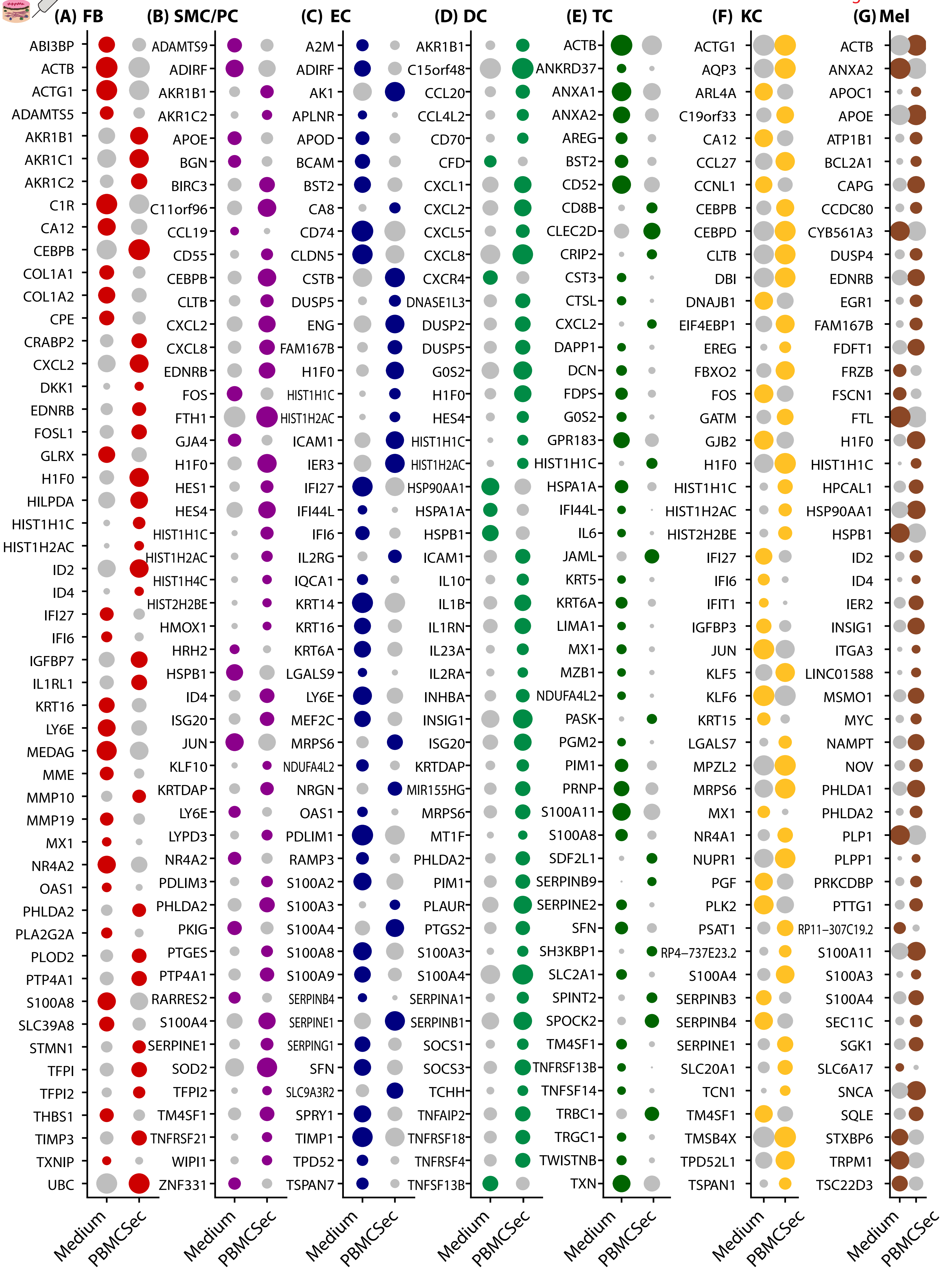

Figure S6: Top 50 regulated genes per cell group in human skin

In A) fibroblasts (FBs, red circles), B) smooth muscle cells and pericytes (SMC/PCs, purple), C) endothelial cells (ECs, blue), D) T-cells (dark green), E) dendritic cells and macrophages (DCs, light green), and keratinocytes (KCs, yellow), melanocytes (Mel, brown); differentially expressed genes (DEGs) were calculated comparing ‘PBMCsec’ skin biopsies to ‘Medium’ skin biopsies, using Wilcoxon rank sum test, including genes with average logarithmic fold change (avglogFC) of > 0.1 or < -0.1 and Bonferroni-adjusted p-value <0.05. For each cellgroup, top 50 DEGs according to lowest adjusted p-value are displayed, split by treatment. Dot size represents percent of cells expressing the respective gene, color correlates with average expression.

Figure S7 scar

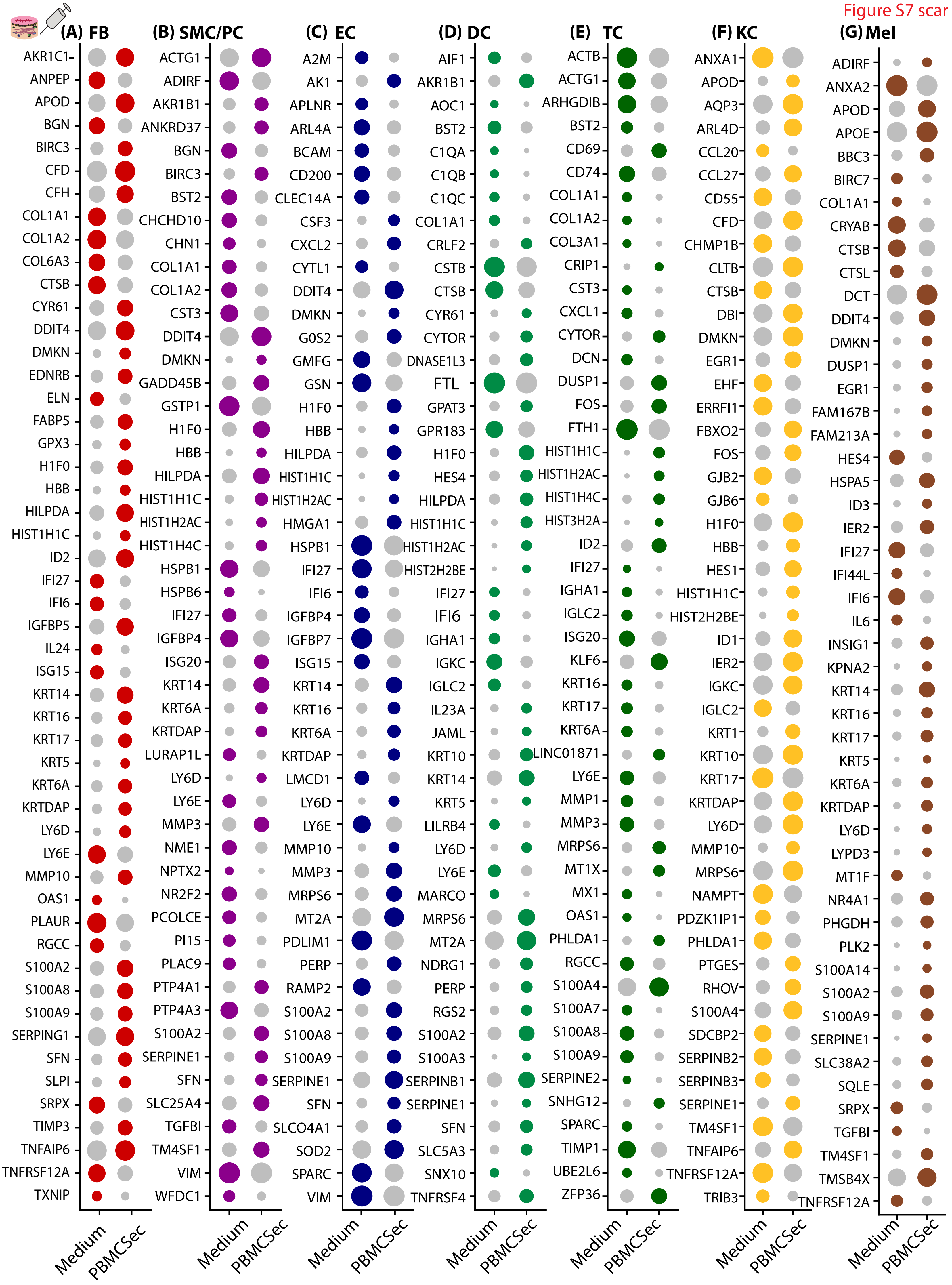

Figure S7: Top 50 regulated genes per cell group in human scar

In A) fibroblasts (FBs, red circles), B) smooth muscle cells and pericytes (SMC/PCs, purple), C) endothelial cells (ECs, blue), D) T-cells (dark green), E) dendritic cells and macrophages (DCs, light green), and keratinocytes (KCs, yellow), melanocytes (Mel, brown); differentially expressed genes (DEGs) were calculated comparing ‘PBMCsec’ scar biopsies to ‘Medium’ scar biopsies, using Wilcoxon rank sum test, including genes with average logarithmic fold change (avglogFC) of > 0.1 or < -0.1 and Bonferroni-adjusted p-value <0.05. For each cellgroup, top 50 DEGs according to lowest adjusted p-value are displayed, split by treatment. Dot size represents percent of cells expressing the respective gene, color correlates with average expression.

Figure S8

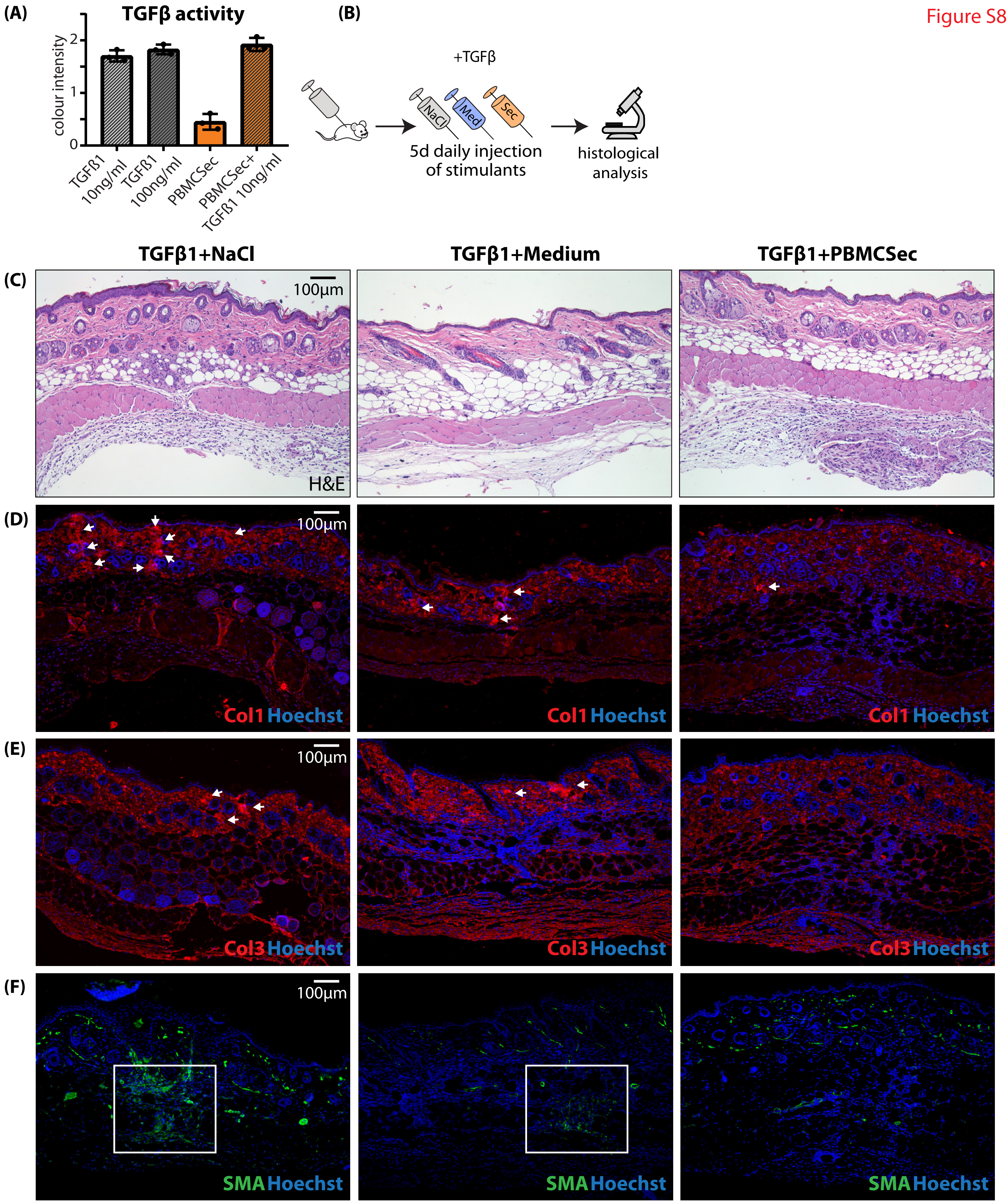
(A)
TGFβ activity
colour intensity
0
1
2
TGFβ1 10ng/ml
TGFβ1 100ng/ml
PBMCSec
PBMCSec+ TGFβ1 10ng/ml
(B)
+TGFβ
NaCl
Med
Sec
5d daily injection of stimulants
histological analysis
TGFβ1+NaCl
TGFβ1+Medium
TGFβ1+PBMCSec
(C)
100µm
H&E
(D)
100µm
Col1 Hoechst
Col1 Hoechst
Col1 Hoechst
(E)
100µm
Col3 Hoechst
Col3 Hoechst
Col3 Hoechst
(F)
100µm
SMA Hoechst
SMA Hoechst
SMA Hoechst

Figure S8: The interaction of TGFβ and PBMCsec *in vivo*

A) In vitro TGFβ-activity assay. HEK-cells colorimetrically detecting SMAD2/3 TGFβ-activity were incubated with recombinant TGFβ1, with PBMCsec alone, and with TGFβ1 and PBMCsec combined. Color intensity correlates with TGFβ1-signaling activity. B) Workflow of mice intradermally injected with TGFβ1 and treatmens. Mice were intradermally injected with 800ng TGFβ1 dissolved 100µl in NaCl 0,9%, Medium or PBMCsec on five consecutive days and sacrificed on the 6th day. C) H&E staining of resulting “scars” of the injected area. Immunofluorescence stainings of D) collagen 1, E) collagen 3, F) smooth muscle actin in TGFβ1-injected mouse skin. Arrows indicate areas of dense matrix deposition.

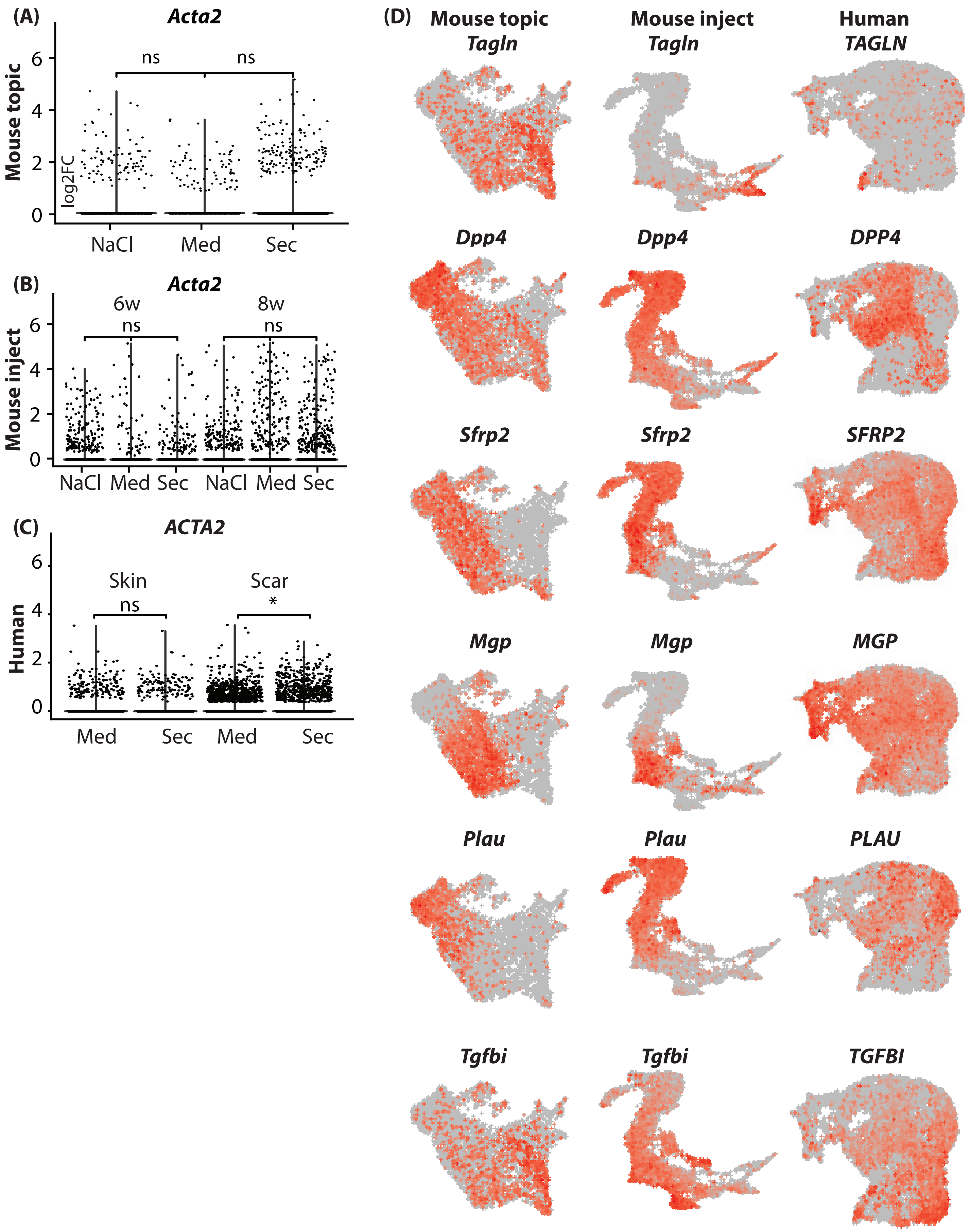
(A)
Acta2
Mouse topic
log2FC
6
4
2
0
ns
ns
NaCl
Med
Sec
(B)
Acta2
Mouse inject
6w
ns
8w
ns
NaCl Med Sec NaCl Med Sec
(C)
ACTA2
Human
Skin
ns
Scar
*
Med
Sec
Med
Sec
(D)
Mouse topic
Tagln
Mouse inject
Tagln
Human
TAGLN
Dpp4
Dpp4
DPP4
Sfrp2
Sfrp2
SFRP2
Mgp
Mgp
MGP
Plau
Plau
PLAU
Tgfbi
Tgfbi
TGFBI

Figure S9: Subcluster analysis of FB populations

A-C) Violin plots of *Acta2/ACTA2* in mouse and human datasets, split by treatments. D) Feature plots of FB subcluster markers in FB subclusters of mouse and human datasets. *Tagln/TAGLN*, Transgelin; *Dpp4/DPP4*, dipeptidyl-peptidase 4; *Sfrp2/SFRP2,* Secreted Frizzled Related Protein 2; *Mgp/MGP,* Matrix Gla Protein; *Plau/PLAU*, urokinase; *Tgfbi/TGFBI*, Transforming Growth Factor Beta Induced. In violin plots, dots represent individual cells, *y*-axis represents log2 fold change of the normalized genes and log-transformed single-cell expression. Vertical lines in violin plots represent maximum expression, shape of each violin represents all results, and width of each violin represents frequency of cells at the respective expression level. In feature plots, normalized log expression of the respective gene is mapped onto the UMAP-plot. Color intensity indicates level of gene expressions. UMAP, uniform manifold approximation and projection. A two-sided Wilcoxon-signed rank test was used in R. NS $p > 0.05$, *$p < 0.05$, **$p < 0.01$, ***$p < 0.001$.
